# Reading for gain or reading for fun: empirical evidence from China on the adoption mechanism of integrated children’s books

**DOI:** 10.3389/fpsyg.2023.1297936

**Published:** 2024-01-12

**Authors:** Jiayong Cui, Jing Guo, Jiaomin Yang, Lingyu Wu, Yinuo Bao

**Affiliations:** ^1^Department of Editing and Publishing, School of Journalism and Communication, Henan University, Kaifeng, China; ^2^Institute of Behavior and Psychology, School of Psychology, Henan University, Kaifeng, China; ^3^Institute of Integrated Publishing Research, School of Journalism and Communication, Henan University, Kaifeng, China

**Keywords:** children reading, integrated publishing, HMSAM, UTAUT, HMS, UMS

## Abstract

**Introduction:**

With the increasing number of children’s publications that integrate new media technologies in the global publishing community, understanding the adoption and use of these publications from a child’s perspective is of great significance for both children’s education practitioners and the publishing industry.

**Methods:**

This article integrates a dual-effect path based on the utilitarian-motivation system (UMS) and hedonic-motivation system (HMS) on information technology adoption via a questionnaire, systematically demonstrating the psychological mechanism of children’s reading with respect to integrated books.

**Results:**

This study finds that children’s willingness to use integrated children’s books is related to UMS and HMS except for facilitating conditions. In addition, this study also reveals the structural differences existing in the adoption psychology of children from various age groups and home education backgrounds.

**Discussion:**

This study provides a systematic explanation for understanding the adoption psychology of integrated children’s books. Based on these findings, it is suggested that publishers should balance the concepts of education orientation and child orientation when producing children’s books, conduct technical innovation of children’s books according to individual children, and constantly innovate the service mode to avoid the risk of children’s bad reading.

## 1 Introduction

Children’s books are an important medium for children and are of great significance to children’s early-stage knowledge acquisition and reading habits cultivation. With the maturity and popularity of digital media technology, children’s book products with increasingly new technological elements based on paper book content continue to bring forth the new through the old and have become a requisite part of the children’s publishing market, bringing an ever-expanding market scale.

There are already numerous cases of new media technology applied to children’s book design around the world. In 2012, a Bologna-based sponsor, in collaboration with the Children’s Technology Review of the United States, launched the ‘BolognaRagazzi Digital Award’ with the aim of producing interactive media products that combine educational and innovative elements from online children’s publications. Since 2018, in the entries, there has been a significant increase in the number of paper books that incorporate technologies such as AR and VR. In 2021, the award was renamed the BolognaRagazzi Cross-Media Award, with a focus on further development and transformation between children’s books and children’s content in other mediums (Bologna Children’s Book Fair, 2021). Thus, it can be seen that the publishing business is not limited to a certain kind of single type of children’s books, such as printing or digital children’s books, and the key focus of the publishing industry has extended to the type of children’s books that integrate multiple media or technologies. This new type of book is different from traditional paper-printed books and is a manifestation of the integrated development of the publishing business. We refer to this new type of book as integrated books.

Existing integrated children’s books mainly reflect two product design concepts: one is to optimize the content presentation, through the implementation of audio and video content, to convey useful messages multimodally and to enhance the readability and expandability of children’s books (Li and Ruiwei, 2017; Children's Literature, 2018; Zhou and Fang, 2023); the other is to improve the user experience of children’s books, to add human–computer interaction such as tap, click, and scanning, to enhance the interactivity and interest of the reading process, and to enhance the charm of products (Huwei et al., 2014; Hu and Jincheng, 2015; Juanjuan, 2017).

Academia generally understands the impact of integrated children’s books on children’s reading matter and education from the perspective of educators or designers. The infiltration of new technologies has made multisensory experiences an important medium for children to absorb knowledge from books. Kljun et al. described and discussed the design space of digitally augmented comic books based on AR technology; Cheng et al. found that students perceive less cognitive load, stronger motivation, and a more positive attitude toward the experience when reading AR books (Cheng, 2017). While grasping children’s attention, multisensory experiences simultaneously enhance their understanding and imagination of book content (Deng and Cong, 2023). Martin et al. explored the learning quality of interactive cooperation among students through different media based on the combinative use of digital reading and textual reading (Kljun et al., 2019).

Past experience in the publishing market shows that the application of new technologies does not always yield positive usage effects and market returns. Especially in the market of children’s books and textbooks, publications adopting new technologies may encounter market resistance due to a variety of factors, such as innovation cost, product pricing, market structure, and educational ideas. Therefore, without a systematic recognition of children’s reading psychology, publishers frequently hesitate to make innovative decisions. Consequently, although a number of publishing houses have tentatively launched new integrated children’s books relying on more advanced new technologies, the mainstream product type in the market is still relatively homogeneous. At the same time, considering that children’s books are purchased by parents for the most part, for the publishing purpose of inculcating knowledge and cultivating individuality, children’s book publishers often ignore the subjectivity of child readers in the process of product design, resulting in the increasingly high proportion of educational attributes in the design process of children’s book products (Duan and Li, 2023). In this regard, the publishing industry has called for the return of the children-oriented concept when publishing children’s books (Zhigang and Fei, 2022). However, there is still a lack of discussion on how to balance the implementation of education-oriented and child-oriented ideas in the children’s book design process.

This article aims to understand the use of integrated children’s books from the perspective of children. As a form of children’s media that applies new media technology, this new media is not only a brand-new educational technology resource but also a novel multimedia technology product. From the perspective of adopting new technologies, the motives of people choosing a new technology product are generally the following: firstly, out of utility considerations, people hope that the new technology can help them better achieve a certain objective. Secondly, for the sake of good feelings, people hope that the new technology can bring unconventional experiences. By utilizing empirical evidence from China, this article explains the psychological mechanism of children’s adoption of comprehensive children’s books, hoping to provide practical and feasible suggestions on innovative product decisions for children’s book publishers. The research issue of this article is: why do children read integrated children’s books? Are there group differences in children’s reading motivation of integrated children’s books?

The purpose of this article is to explore the psychological mechanisms of children’s adoption of integrated children’s books through empirical evidence from the perspective of technology adoption and, then, to provide practical suggestions for children’s book publishers to make decisions on product innovation.

## 2 Literature review and research hypothesis

### 2.1 Utilitarian motivation and hedonic motivation: dual mechanisms for the adoption of integrated children’s books

The rapid development of digital media technology has triggered the digital transformation of the publishing business, with more and more integrated publications subsequently emerging. In 2017, a product called *Tara’s Locket*, developed by the Big Motive Digital Product Innovation Studio in Ireland, which combines VR technology with graphic content, was nominated for the *BolognaRagazzi Digital Award*. In 2017, the Quantum Story Company launched a series of children’s books called *‘Operation YOU’*, which combines traditional printing techniques, augmented reality (AR), and virtual reality (VR). Moreover, through a free smartphone app, printed text can fly off the paper and be presented in a highly interactive and visual form for readers. In addition, tailored VR glasses can also be used to allow readers to see vivid and wonderful virtual images. In a word, the emergence of integrated publication has sparked interest for all concerned in explaining its adoption motivations in both publishing circles and academia.

In terms of the explanation of adoption motivation, most technology adoption studies focus on pragmatic motives, which reflect users’ practical purposes for the determination of new technology adoption. For example, Vasiuk et al. (2022) argue that ‘learning knowledge’ and ‘mastering professional skills’ are the main motives for students to study (Vasiuk et al., 2022). Lin et al. (2023) hold that the utilitarian benefits (i.e., being helpful, effective, functional, necessary, and practical) that AI-based social robots bring to children’s English learning are of great significance for the adoption rate of AI-based social robots (Lin et al., 2023). Al-Abdullatif and Alsubaie (2022) argue that behavioral intentions and facilitating conditions are significant determinants of teachers’ actual use of the IRA digital platform. Chin et al. (2020) assume that the most influential factors in the consumptive use of enterprise social network are content value and performance expectancy. Fagan (2019) reckons that performance expectancy has a prominent and direct effect on the intention to use iPads for m-learning.

Correspondingly, there is another intrinsic emotional motivation toward the product itself. For example, Hamari and Koivisto (2015) found that hedonic motivations have a positive direct association with continued use. Aboelmaged (2018) believe that hedonic motivations have a positive profound influence on using ESN for knowledge sharing (Aboelmaged, 2018). Fagan (2019) found that the total effect of hedonic motivation on intention was significant, while the effect of hedonic motivation on intention was fully mediated by performance expectancy. Song et al. (2015) argue that utilitarian and hedonic expectations had a positive impact on users’ intentions to adopt 3G mobile technology. Oluwajana et al. (2019) believe that the acceptance of a gamified learning environment could serve as a new educational tool to expedite the improvement of pedagogical and instructional technology.

It can be seen that academia has outlined two main interpretation paths for studying the motivation of information technology adoption, namely, whether users primarily use technology out of utilitarian or hedonic purposes. Based on traditional paper-based children’s books, integrated children’s books construct a complex type of information system by introducing new presentation forms and interaction techniques. Technology is an essential component of information systems, providing the base and support for information systems. Conversely, information systems are also an important stage for technological applications. Therefore, this article takes technology adoption motivation as the theoretical basis for the adoption of integrated children’s books. Indeed, theoretical models of relevant information system adoption research can be used to explain two psychological mechanisms of integrated children’s book adoption: utilitarian motivation and hedonic motivation. They are constructed as two classic technology adoption models: the **Unified Theory of Acceptance and Use of Technology (UTAUT)** and the **Hedonic-Motivation System Adoption Model (HMSAM)**. These are the theoretical starting points for constructing a systematic interpretation model for this study.

The UTAUT is mainly used to explain the influential mechanism of utilitarian motivation on technology adoption, and it is composed of eight theoretical models, including the Theory of Reasoned Action, Technology Acceptance Model, and Motivation Model. These models contain four core exogenous latent variables and four exogenous moderating variables, which are used to explain users’ usage intentions and usage behaviors (Venkatesh et al., 2003). The UTAUT and its topology model have been used to explain information system adoption behaviors in a variety of contexts, such as mobile health, mobile wallet, and mobile payment (Dwivedi et al., 2020), where the exogenous variables of performance expectancy, effort expectancy, community influences, and facilitating conditions and the endogenous variables of usage intention and usage behavior are the six most frequently used core variables.

The HMSAM is primarily used to explain the mechanisms by which hedonic motivation influences technology adoption. In light of the recent boom of social networks and video games, Lowry argues that attention should not only be focused on the role of **utilitarian-motivation systems (UMS),** which are a specific outcome of information system use, but also on how **hedonic-motivation systems (HMS)** play a role in the usage process itself. Therefore, Lowry constructed the HMSAM to emphasize the explanation of HMS mechanisms. In addition to performance expectations (perceived usefulness), the HMSAM places more emphasis on the influence of effort expectations (perceived ease of use), curiosity, and joy on users’ usage intentions. The HMSAM has been used in relevant information system adoption research, such as online education (Francke and Alexander, 2018; Rosmansyah et al., 2019), radio programs (Lim and Park, 2016), mobile health (Lim and Park, 2016), VR travel (Kim and Hall, 2019), and online shopping (Xiaozhou, 2020), and its validity has been preliminarily verified.

This article argues that children’s willingness to read integrated children’s books may be collectively determined by utilitarian motivation and hedonic motivation. Therefore, this study integrates the two underlying models, the UTAUT and HMSAM, in order to validate the psychological mechanisms of children’s adoption of integrated children’s books.

### 2.2 The effect of hedonic motivation on reading intentions for integrated children’s books

Curiosity and joy are two key variables in the HMSAM that explain users’ adoption intentions. In studies on information system adoption, users’ curiosity was found to enhance their usage intention (Qin et al., 2009). According to educational psychology, curiosity is a kind of aspiration of children for intrinsic development, and curious young children are more creative and more willing to attempt and discover (Liu, 2004). Therefore, this article defines ‘curiosity’ as the extent to which children are curious about integrated children’s books and hypothesizes:

*H1a: Children’s curiosity about integrated children’s books has a significant positive effect on their intention to read.*

Joy refers to, in addition to the expected utility, the degree of pleasure and satisfaction for users brought by the process of using an information system (Hong and Tam, 2006). User studies from certain mobile social media have found that enjoyment has a significant impact on users’ usage intentions and usage behaviors (Kim and Forsythe, 2008). Educational psychology believes that game-based learning is conducive to stimulating students’ learning pleasure, guiding them to gain new knowledge and improve their academic performance (Lijuan, 2007). Research on children’s educational apps has found that most apps would optimize their character settings, interface design, storylines, and voice-overs to increase the interest of the reading content to meet the demand of “teaching for fun” (Zheng and Zhuang, 2017). Therefore, this article defines ‘joy’ as the fun of integrated children’s books that can be perceived by children and assumes that:

*H2a: Children’s joy perception of integrated children’s books has a significant positive effect on their intention to read.*

### 2.3 Influence of utilitarian motivation on reading intentions of integrated children’s books

Utilitarian motivation in the UTAUT model consists of four key variables: performance expectancy, effort expectancy, social influence, and facilitating conditions. **Performance expectancy (PE)** refers to the assistance extent for users’ work that is brought by new technology, and this variable is also referred to as perceived usefulness in certain models such as TAM. In the context of home education, parents’ performance expectancy - i.e., the help provided for children’s learning - can also have an impact on children’s behavioral intentions to learn. For example, it has been found that primary school students’ home motivations for learning English are mainly instrumental, and few parents believe that learning English will not benefit their children (Yutong, 2021) In the reading context, empirical studies on online reading (Liu and Liu, 2015) and audiobooks (Zhipeng and Zhang, 2020) have shown that the performance expectation is an important variable influencing readers’ usage intentions. Research on children’s digital reading has also found that parents’ perceived usefulness of digital publications had a significant effect on children’s digital reading intentions (Han, 2019). Therefore, this article defines ‘performance expectation’ as whether children think that integrated children’s books can help them gain knowledge and competence and hypothesizes:

*H3a: Children’s performance expectations for integrated children’s books have a significant positive effect on their intention to read.*

**Effort expectancy (EE)** is the difficulty of using a system. In TAM, MPCU, IDT, and other models, effort expectancy is also called perceived ease of use. Some studies have suggested that tablet-based electronic picture books are more nearly three-dimensional and interesting in shaping characters, and the audiovisual effects can provide children with more intuitive image shaping, and child readers are therefore more likely to apply reality to the story characters (Wei and Juan, 2017). The results of relevant empirical studies show that the higher the perceived ease of use of an online reading environment (Venkatesh et al., 2003) and e-reading devices by children (Rosmansyah et al., 2019), the stronger their reading intentions. Therefore, this article defines ‘effort expectancy’ as children’s difficulty in using integrated children’s books and hypothesizes:

*H4a: Children’s effort expectancy of integrated children’s books has a significant positive effect on children’s reading intentions.*

**Social impact (SI)** is derived from the subjective norms and social factors of the Theory of Planned Behavior, which refers to the degree to which individuals are influenced by surrounding groups. Some researchers have pointed out that social impact does not have a significant effect on the usage willingness of information system users in the case of voluntary use (Wu, 2016). However, in the study of home education, some researchers have pointed out that parents sometimes tend to compare with higher achievers, thus struggling to create ideal living and learning conditions for their children (Wu, 2009). With the emergence of education marketization, the comparison mentality of parents has become increasingly complicated, leading to irrational education expenditure and even poverty caused by ‘education’ (Jiuyu, 2019). Moreover, kids also have a certain degree of comparison, which is caused by the adult-oriented social environment in which teachers and parents are involved (Jiaqi, 2017). Therefore, this article defines the macro sense of ‘community influence’ as children’s subjective perception of the community influence of reading integrated children’s books and hypothesizes:

*H5a: Children’s perceptions of community influence of integrated children’s books have a significant positive effect on reading intentions.*

**Facilitating conditions (FCs)** refer to the individuals’ possession of knowledge and resources that are available for using a new technology or system. The effect of facilitating conditions on users’ usage willingness has been preliminarily verified in previous research on information system usage behaviors (Si-Hang, 2018). In the educational context, some studies have found that facilitating conditions can explain users’ adoption intentions and usage behaviors of new educational technologies when they use electronic educational devices (Qin et al., 2018) and online educational resources (Geng, 2016; Chao and Mei, 2022). This article defines ‘facilitating conditions’ as technological or resource support that children can access when reading integrated children’s books and hypothesizes:

*H6a: Facilitating conditions have a significant positive effect on children’s behavioral intention to use integrated children’s books.*

In addition, it has been found that in the UTAUT model, facilitating conditions not only indirectly affect users’ usage behaviors by influencing users’ usage intention but also directly affect users’ usage behaviors. Therefore, this article hypothesizes:

*H7: Facilitating conditions have a significant positive effect on children’s reading behavior of integrated children’s books.*

### 2.4 The mediation role of reading intentions of integrated children’s books

**Behavioral intention of usage (BIOU)** is the intensity of a user’s willingness to continue to use an information system (Venkatesh and Davis, 2000). **Behavior of usage (BOU)** is the behavior of users who continue to use technology and recommend it to their friends and relatives after the initial trial of the technology (Fishbein and Ajzen, 1977). In studies on online education (Li, 2016) and the usage behavior of open educational resources in universities (Kim and Forsythe, 2008), scholars have found that usage intention has a strong influence on usage behavior. Therefore, this article assumes that children’s reading intentions of integrated children’s books have a direct influence on their reading behavior. Therefore, this article hypothesizes:

*H1b to H6b: Children’s behavioral intention to use integrated children’s books has a significant positive effect on their behavior in using children’s books.*

**Figure 1:**
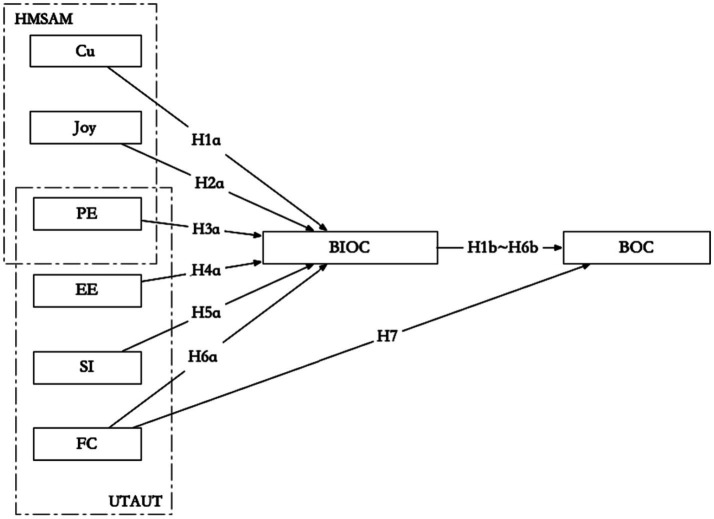
Theoretical hypothesis model for the adoption of integrated children’s books.

In summary, this article constructs an integrated theoretical model of integrated children’s books adoption behavior based on the HMSAM and UTAUT, as shown in Figure 1.

## 3 Methodology

### 3.1 Measurements

The scales required for this study were altered based on the conceptual interpretations in the UTAUT and HMSAM, with reference to well-established measurement tools in the relevant literature. A total of eight concepts were included in the model for measurement, and the literature sources and the scale items in the formal survey are shown in Table 1. The items were measured using a five-point Likert scale, with numbers 1 to 5 indicating ‘not at all’, ‘not quite’, ‘maybe’ ‘comparatively conform’, and ‘completely conform’. In the questionnaire, the items appear in random order.

**Table 1:** Scale design and literature sources.

**Concept**	**Question**	**Scale source**
Curiosity	Cu1 My children are always very curious when they see ‘new-type children’s books’.	Agarwal and Karahanna (2000) and Lowry et al. (2012)
	Cu2 ‘New-type children’s books’ will stimulate my child’s imagination.	
	Cu3 ‘New-type children’s books’ have always aroused my child’s curiosity.	
Joy	J1 My children can discover fun in ‘new-type children’s books’.	Agarwal and Karahanna (2000) and Lowry et al. (2012)
	J2 My kids are happy when they read ‘new-type children’s books’.	
	J3 My children are very happy when they read ‘new-type children’s books’.	
Performance expectancy PE	PE1 Children think that they can learn knowledge quickly with ‘new-type children’s books’.	Davis (1989) and Venkatesh et al. (2003)
	PE2 Children think that ‘new-type children’s books’ will help them learn more.	
	PE3 Children think that reading ‘new-type children’s books’ is very efficient.	
	PE4 Children think that ‘new-type children’s books’ make reading relaxing.	
Effort expectancy EE	EE1 Children find that ‘new-type children’s books’ are easy to use.	Davis (1989) and Venkatesh et al. (2003)
	EE2 Reading ‘new-type children’s books’ is easy for children.	
	EE3 Children find it is easy and simple to read new-type children’s books.	
Social impact SI	SI1 My child also wants to have one when they see other children reading ‘new-type children’s books’.	Moore and Benbasat (1991) and Venkatesh et al. (2003)
	SI2 Children believe that many children are reading ‘new-type children’s books’ nowadays.	
	SI3 Children feel that reading ‘new-type children’s books’ improves other children’s abilities or knowledge.	
Facilitating conditions FCs	FC1 My child has the necessary equipment to read ‘new-type children’s books’, such as reading pens, electronic reading machines, mobile phones, and tablets.	Thompson et al. (1991) and Venkatesh et al. (2003)
	FC2 My child has sufficient knowledge or skills to read ‘new-type children’s books’.	
	FC3 My child has access to ample network resources if necessary, such as mobile traffic and wireless Wi-Fi.	
	FC4 ‘New-type children’s books’ have simple instructions that children can understand at a glance.	
	FC5 When the going gets tough, there is always someone who can teach a child how to use ‘new-type children’s books’.	
Behavioral Intention of Usage BIOC	BIOC1 My children really enjoy reading ‘new-type children’s books.’	Bhattacherjeen (2001)
	BIOC2 My children always want to read ‘new-type children’s books’.	
	BIOC3 My children invite a small group of friends to read ‘new-type children’s books’.	
Behavior of usage BOC	BOC1 My child regularly reads ‘new-type children’s books’.	Venkatesh et al. (2003), Kankanhalli et al. (2005) and He and Wei (2009)
	BOC2 My child frequently reads ‘new-type children’s books’.	
	BOC3 My child spends a lot of time on ‘new-type children’s books’.	

### 3.2 Data collection

In July 2022, we distributed the questionnaire through Questionnaire.com. The respondents were informed of the purpose and process of the survey to confirm that they were voluntarily participating in the questionnaire survey with full informed consent. Certainly, they had the right to withdraw from the survey at any time during the process. The questionnaire screened the object of the study through two questions, the first being whether there were children under 12 years of age in the household and the second being whether they had seen or come into contact with integrated children’s books.^1^ A pre-survey was conducted prior to the formal survey in order to validate and correct the scale (*n* = 190). Taking into account the potential ethical risk existing in juvenile research, the questionnaires were completed by the parents on behalf of their children. The research process was examined by the School of Journalism and Communication, Henan University (approval number: 2023–0001).

The formal survey was based on the data of the age distribution of the regional population in the *China Statistical Yearbook 2021* (National Bureau of Statistics of China, 2021), and the sampling frame was delineated using regional stratified sampling. The regional distribution is shown in Table 2. A total of 513 questionnaires were collected in the formal survey, and 334 valid questionnaires were screened. The average time to complete a valid questionnaire was 5 min and 56 s. The average annual household income of the respondents was 162,000 yuan (SD = 20.807), the average age of the parents was 36.1 years (SD = 5.825), the education level of the parents was mainly ‘undergraduate/diploma’ (*n* = 168), and there were more female parents who completed the survey (*n* = 240). The average age of the children was 7.8 years (SD = 3.500), with the proportion of girls (48.2 per cent) being slightly lower than that of boys. The sample was essentially in line with the population distribution characteristics of Chinese adolescents.

**Table 2:** Regional distribution of respondents.

Region	Population (0–14)	Sample	Region	Population (0–14)	Sample	Region	Population (0–14)	Sample
Beijing	2,591,507	3	Anhui	11,742,682	15	Chongqing	5,098,363	7
Tianjin	1,868,056	2	Fujian	8,025,225	11	Sichuan	13,471,112	18
Hebei	15,088,968	20	Jiangxi	9,922,364	13	Guizhou	9,242,038	12
Shanxi	5,709,895	8	Shandong	19,062,638	25	Yunnan	9,237,474	12
Inner Mongolia	3,377,673	4	Henan	22,988,954	31	Tibet	894,865	1
Liaoning	4,737,939	6	Hubei	9,420,477	12	Shaanxi	6,852,205	9
Jilin	2,818,723	4	Hunan	12,969,522	17	Gansu	4,853,543	6
Heilongjiang	3,286,466	4	Guangdong	23,749,882	31	Oinghai	1,232,956	2
Shanghai	2,436,296	3	Guangxi	11,842,501	17	Ningxia	1,468,004	2
Zhejiang	12,891,948	17						

### 3.3 Data analysis

In this article, SPSS26 was used for the pre-processing of the questionnaire, descriptive statistics of variables, and reliability and validity tests. The process plug-in was used for the mediating effect test. Simultaneously, the AMOS software was employed to construct a structural equation model and to subsequently perform analysis and group comparisons.

## 4 Data analysis and research findings

### 4.1 Reliability test of the scales

The internal consistency reliability (Cronbach’s alpha) and combined reliability (CR) were calculated for the scales, as shown in Table 3. The internal consistency (alpha) reliability of the conceptual scales was entirely greater than 0.7 in the pre-survey and the formal survey, and the combined reliability (CR) was entirely greater than 0.8, indicating that the reliability of the scale is generally good. The structural validity of the scales was calculated by the method of confirmatory factor analysis, extracting a common factor stably from each scale. The factor loading and KMO of each question item were greater than 0.6, and the AVE was greater than 0.7, indicating that the structural validity of the scales was generally good.

**Table 3:** Reliability test results of each scale.

	Pre-survey (*n* = 334)			
	α	CR	KMO	AVE
Cu	0.813	0.890	0.815	0.854
Joy	0.881	0.928	0.884	0.900
PE	0.878	0.917	0.845	0.856
EE	0.808	0.887	0.868	0.850
SI	0.700	0.833	0.879	0.789
FC	0.703	0.808	0.808	0.676
BIOC	0.839	0.905	0.676	0.872
BIC	0.867	0.919	0.693	0.889

The AVE square root and correlation coefficient of the scales were calculated to test discrimination among the scales, as shown in Table 4. The correlation coefficient among the scales was entirely less than the AVE square root, indicating that the scales had good discrimination.

**Table 4:** Pearson correlation coefficient and AVE mean value for the scales.

	Cu	Joy	PE	EE	SI	FC	BIOC	BIC
Cu	0.924							
Joy	0.822**	0.949						
PE	0.726**	0.662**	0.925					
EE	0.637**	0.569**	0.827**	0.922				
SI	0.676**	0.615**	0.705**	0.683**	0.888			
FC	0.570**	0.515**	0.584**	0.567**	0.683**	0.822		
BIOC	0.602**	0.539**	0.683**	0.633**	0.736**	0.616**	0.934	
BIC	0.482**	0.428**	0.511**	0.498**	0.639**	0.688**	0.615**	0.943

** denotes *p* < 0.01, and values on the diagonal line are the AVE mean value of the scale.

### 4.2 Structural equation modeling analysis

**Figure 2:**
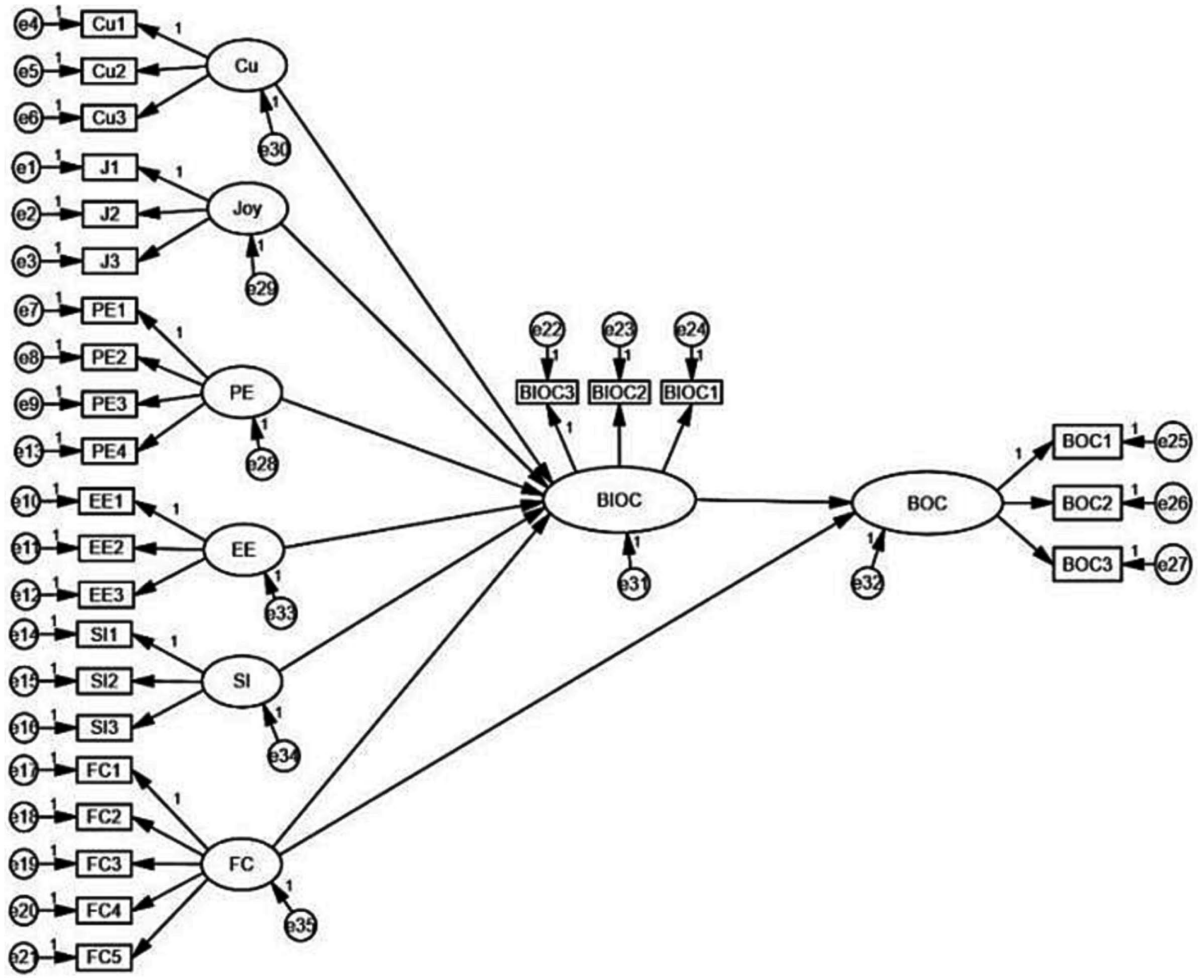
Initial structural equation model.

It was necessary to construct a structural equation model based on the theoretical model. The initial model contained a total of eight latent variables and eight effect paths among the latent variables, as shown in Figure 2. The results of the initial analysis showed that the effect paths of curiosity, joy, performance expectation, effort expectation, and social influence on children’s reading intentions were significant in the structural model, the effect path of facilitating conditions on children’s reading intentions was not significant (*γ* = 0.024, *p* = 0.708), and the effect path of facilitating conditions on children’s reading behaviors was also not significant (*γ* = 0.073, *p* = 0.299). Therefore, the initial model needed to be corrected.

**Figure 3:**
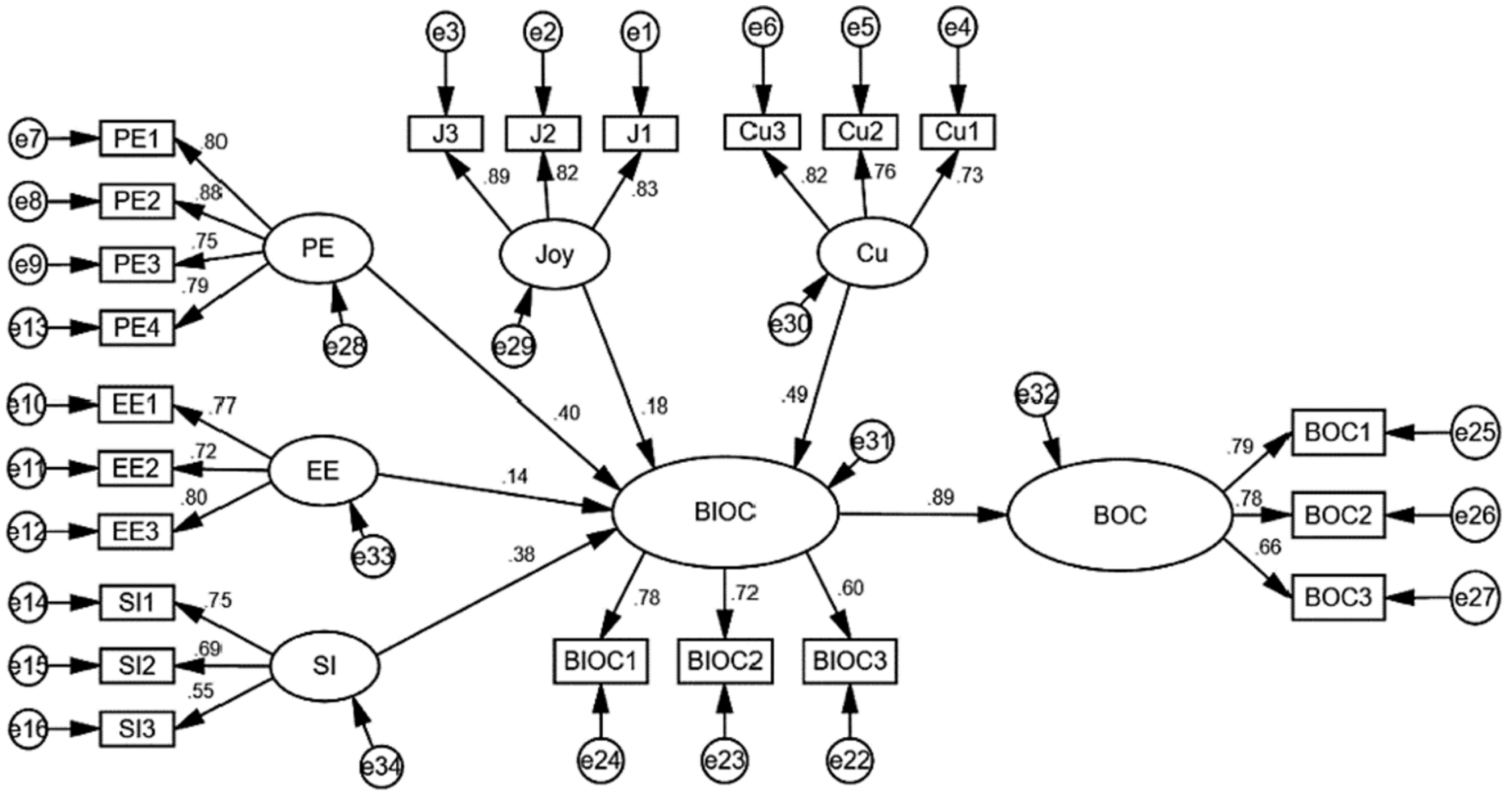
Modified structural equation model 2.

After deleting the insignificant structural model path, the modified structural equation model was obtained, as shown in Figure 3^2^. After testing, each fit index of the modified model conformed to the determination conditions: since the chi-square value was greatly affected by the number of parameters and the number of samples (Qiu and Lin, 2009), the relative fit index (GFI = 0.910, AGFI = 0.880, PGFI = 0.684), the substitutability index (CFI = 0.970, RMSEA = 0.049), and the residual analysis of the test model (RMR = 0.033, SRMR = 0.039) were used to test the fitting of the model. Except for AGFI, which was slightly lower than the standard criterion, all the fit indices were within the scope of judgment (Qiu and Lin, 2009), indicating that the model had good explanatory power.

The revised structural equation model showed that the effect paths of curiosity, joy, performance expectations, effort expectations, and community influence on children’s reading intentions were generally significant, and the effect path of children’s reading intentions on children’s reading behaviors was also generally significant. Therefore, hypotheses H1a ~ H5a and H1b ~ H5b were preliminarily supported, while H6a, H6b, and H7 were not valid.

### 4.3 Mediation effect test

Based on the structural equation model, a further mediation effect test was performed. The latent variables in the structural model were firstly packaged with the factor methods (Yan and Wen, 2011), and then, we used the bootstrap method to perform 5,000 instances of repeated sampling and to analyze the mediation effects of the structural model through the controls of child gender, child age, parental educational level, and annual household income.

As shown in Table 5, the test results showed that the mediation effect of effort expectation (EE) on the behavior of children’s reading (BOC) through the behavioral intention of children’s reading (BIOC) was not significant due to the fact that the first half of the pathway was not significant (*β* = 0.077, *p* = 0.175), suggesting that the pathway was not sound in the structural equation model (further explanation will be provided below through group comparisons). The mediation effect of the other four paths were all significant (none of the confidence intervals contained 0), and hypotheses H1, H2, H3, H4b, and H5 were supported. The results of the mediation effects test also showed that the control variables of child age (*β* = −0.023*) and parental educational level (*β* = −0.077*) also had a significant effect on children’s reading behavior.

**Table 5:** Summary of the results of the mediation effects test (*n* = 334).

trails	c	a	b	a*b	a*b Boot CI
Cu= > BIOC= > BOC	0.218**	0.294**	0.671**	0.197	0.103 ~ 0.301
Joy= > BIOC= > BOC	0.059	0.145*	0.671**	0.097	0.010 ~ 0.186
PE= > BIOC= > BOC	0.258**	0.180**	0.671**	0.121	0.026 ~ 0.223
EE= > BIOC= > BOC	0.110*	0.063	0.671**	0.042	−0.027 ~ 0.112
SI= > BIOC= > BOC	0.207**	0.247**	0.671**	0.166	0.084 ~ 0.254

* denotes *p* < 0.05, ** denotes *p* < 0.01.

### 4.4 Group comparison

Since the results of the mediation effect test slightly differed from the structural equation model after the inclusion of the control variables, the group comparison of the structural equation model was conducted based on the differences of children’s age and parents’ educational level. The children were divided into three groups according to their age distribution: old age (10–12 years old), middle age (7–9 years old), and young age (0–6 years old); the parents were divided into three groups according to their educational level: high level (master’s degree and above), middle level (university or diploma), and low level (senior high school and below). The results of the group comparison are shown in Table 6.

**Table 6:** Summary of standardized path coefficient for each subgroup in the structural model.

trails	Age of the child			Parents’ level of education		
	young (*n* = 115)	Medium (*n* = 105)	old (*n* = 114)	Low (*n* = 75)	Medium (*n* = 168)	High (*n* = 91)
Joy= > BIOC	0.131	0.025	0.293**	0.607***	0.088	0.042
Cu= > BIOC	0.644***	0.624***	0.253*	−0.013	0.515***	0.814***
PE= > BIOC	0.191*	0.445***	0.528***	0.386**	0.439***	0.231*
EE= > BIOC	0.316**	−0.007	0.064	−0.008	0.305***	0.192*
SI= > BIOC	0.318**	0.288**	0.422**	0.185	0.321***	0.230*
BIOC= > BOC	0.896***	0.95***	0.822***	0.945***	0.864***	0.885***

* indicates *p* < 0.05, ** indicates *p* < 0.01, *** indicates *p* < 0.001.

From the path coefficient of the structural model for each child age subgroup, there was detected a certain influence of joy, curiosity, performance expectancy, and effort expectation on the adoption mechanism of integrated children’s books. As children grow older, the effects of curiosity and effort expectation on children’s reading intentions gradually decline, while the effects of joy and performance expectancy on children’s reading intentions become increasingly strong.

In terms of family educational background, the effect path of hedonic motivation on children’s reading intentions was potentially constrained by the level of parental education. In the low-level group (parents’ educational level at senior high school level and below), joy was found to be an important factor driving children’s reading intentions, which was not significant in any of the medium-level or high-level groups. In contrast, the role of curiosity in children’s reading of integrated children’s books was found to become more prominent as the parents’ educational level enhanced.

## 5 Results

**Figure 4:**
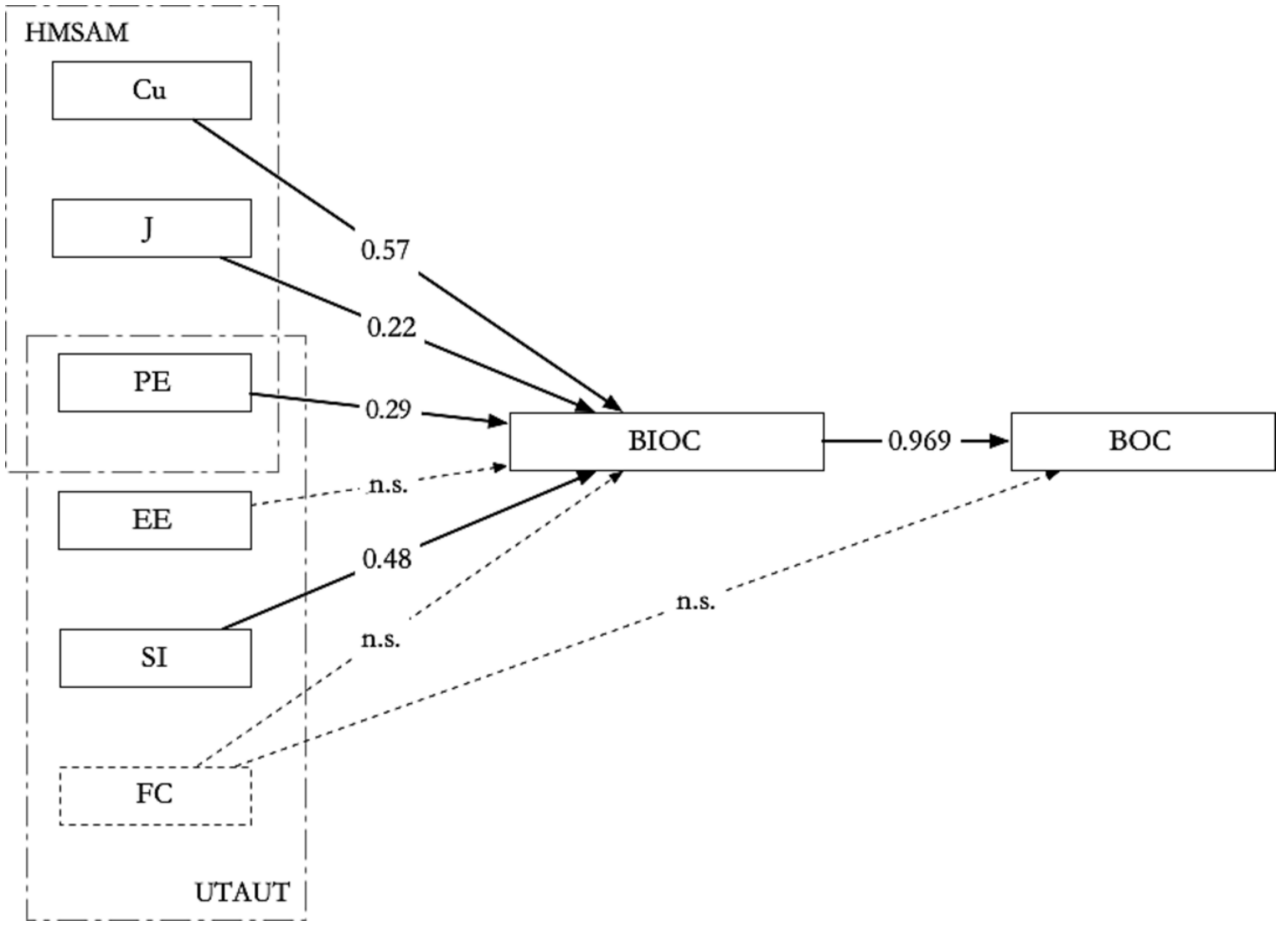
Validated convergent children’s books adoption model.

Based on the UTAUT and HMSAM, this article has constructed and validated an adoption model for integrated children’s books, as shown in Figure 4.

The model tests and verifies the assumption from previous texts that integrated children’s book reading is influenced by both hedonic motivation and utilitarian motivation. The two existing technological concepts of integrated children’s books also indicate that designers hope new technology could not only help children understand and grasp knowledge but also raise their interest in reading. It is conducive to achieving the goal of ‘teaching for fun’. In addition, the data analysis reveals that the factors influencing the adoption of integrated children’s books may differ among children of different ages and home educational environments, which provides empirical evidence for technological innovation and the publishing marketing of children’s book products.

In the aspect of hedonic motivation, the data tests the positive impact of curiosity and joy on children’s reading intentions and reading behaviors in general. For children, the new technique elements of integrated children’s books make the reading experience more attractive and interesting, which contributes to the cultivation of their reading habits. The results of the data analysis also show that the impact of joy and curiosity on children’s reading intentions is varied across different age groups and home educational environments. For younger children, the emergence of new technology may make them itch to try it out of curiosity, but they are not yet ready to appreciate the joy of reading. As children get older, they lose their curiosity about integrated books, and only those who enjoy reading integrated books will continue to use them. Curiosity is less important for children whose parents have lower levels of education, and only those who can perceive the pleasure of reading will have a higher intention to read.

In the aspect of utilitarian motivation, the data tests the effects of performance expectations, effort expectations, and community influence on children’s reading intentions in general. As children grow older, the influence of reading performance expectations on children’s reading intentions becomes more prominent, while the influence of effort expectations on their reading intentions diminishes. For children of any age, community influence is a critical factor in their choice of integrated children’s books. This suggests that there is also a group effect on children’s reading and that herd mentality and mind of rivalry will reinforce children’s reading intentions, which is a reflection of children’s early socialization. The effect of facilitating conditions on reading intentions was not supported by the data. In the original UTAUT model, the researcher also found that facilitating conditions could not affect users’ usage intentions as a separate explanatory variable, but they work together with other variables such as age and use experience. Only elderly users who are not familiar with technology need a service guide for an information system (Zheng and Zhuang, 2017). This article argues that because China has made great achievements in information infrastructure construction, the Internet and electronic equipment have already entered thousands of households; at the same time, the technical solutions of the integrated children’s book products sold in the market today are relatively simple, and children often do not need to rely on product usage instructions or support services when they use such products.

## 6 Discussion

Based on the empirical evidence provided by empirical studies for technological innovation and the publishing marketing of children’s book products, this article argues that children’s book publishers can optimize the publishing progression of integrated children’s book products in the following three dimensions.

### 6.1 Balance between the concepts of education orientation and child orientation

Driven by the reading concept of ‘Reading is always beneficial’, parents naturally hope that their children can acquire a certain amount of knowledge from children’s books, and publishers also attach great importance to the educational attributes in integrated children’s books when designing and selling them. However, from the perspective of children’s psychological development law, children’s early psychological characteristics are curiosity of the perceptual world, and utilitarian goals such as the acquisition of knowledge and the perceived utility require a certain amount of accumulation of social experience. This means that it is difficult for children to internalize their parents’ utilitarian reading motivations at an early age. The empirical evidence from this study also suggests that, at an early age, children are insensitive to the educational functions of integrated children’s books and lack cognitive awareness of the delight of reading because their willingness to read integrated children’s books is mainly driven by curiosity. This finding reminds children’s book publishers that they should pay extra attention to ‘child orientation’, i.e., whether the product is appealing to children when designing integrated children’s books for younger children.

However, this article argues that the design of integrated children’s books must not ignore educational factors completely. According to Piaget’s explanation of the stages of children’s psychological development, children aged 2 to 7 are in a critical period of early socialization (Piaget, 1982). The empirical evidence provided in this article also suggests that children’s perception of the utility of reading will be gradually strengthened as they enter the school-age stage. If children’s books do not help children develop good reading habits, it is feared that school-age children will find it difficult to develop an interest in reading. Publishers should pay attention to the balance between the concepts of child orientation and education orientation when pursuing integrated children’s book products with long life cycles: not only should integrated children’s books conform to children’s nature of strong curiosity and love of fun at a very young stage, but they should also allow children to capture the joy of reading progressively and help them develop good reading habits so that they can also gain knowledge through reading when they are of school age.

### 6.2 Technology-enabled children’s book publishing needs to be child specific

In the discussion of children’s reading, there is no lack of discussion on the issue of level reading, but studies have mainly been concerned with ‘how to read’, such as the analysis of children’s reading catalogues launched by European and American booksellers or the children’s book scoring table that elucidates the reading levels of Chinese children (Hongwei, 2010). The report called *Opinions on Promoting Reading for All issued by the Central Propaganda Department in 2020* stated that ‘carrying out activities compatible with the physical and mental development of preschoolers and conducive to the cultivation of reading interests and reading habits’ is an important task in the course of nationwide reading that should target all young people. Within the social background of a highly digitized media environment, publishers should also attach importance to the reading psychology of children of different ages when launching integrated children’s book products and pay more attention to the issue of ‘how to read’.

Based on the empirical evidence provided in the previous section, publishers can adopt targeted technology empowerment strategies according to the reading psychology of children of different ages when designing integrated children’s book products. For younger children aged 0–6, publishers should pay extra attention to the design of sound, animation, and other content elements to attract their perennial attention and usage as they are still in the early stage of socialization. For middle and older children aged 7 and above, due to the maturity of their minds, the low threshold of interaction and relatively simple content design may be a bit childish. Publishers can contribute to enriching the usage experience of their products with more advanced content presentation technologies, such as VR and AR, and utilize interaction techniques that require a higher degree of operational precision to attract middle-aged children and older children to use their products. Meanwhile, publishers also need to gradually strengthen the knowledge attributes of their content step by step to meet the knowledge needs of middle-aged children and older children.

### 6.3 Optimizing product service patterns to avoid the risk of children’s bad reading

Proponents of the parent–child reading model believe that parental companionship and guidance are key to influencing children’s reading efficiency. However, in the real world, not all parents possess the concept of parent–child reading, nor do they have the ability or time to accompany their kids in reading. Moreover, according to the empirical evidence provided in this article, curiosity, playfulness, and community imitation may become new motives to promote children’s reading after adding new technology elements to integrated children’s books. Especially in subgroups where parents have low education levels, product enjoyment has a greater impact on children’s willingness to use them. Therefore, it is difficult to ensure that such integrated children’s book products can be used appropriately. The recent incident of pornographic content on the ‘Little Genius Tablet’ is also an alarm bell for the design and regulation of children’s educational products. Publishers must therefore consider how to regulate the use of their products to avoid the risk of children’s bad reading.

This article argues that children’s book publishers can avoid the occurrence of similar risky events through product and service innovation. First, they can provide parents and children with value-added educational services such as reading guidance. Currently, there are some children’s accompanied reading apps in the market based on the common needs of parents and children that can design reading topics according to the ideas of children and parents. During their children’s reading process, parents will also receive reading instructions or content interpretation reminders. When designing integrated children’s book products, publishers can also expand the content appropriately based on the needs of parent–child reading so that children’s books can play an educational function and become a bond that maintains parent–child relationships as well.

The second is to strengthen the control of children’s reading processes to help them cultivate healthy reading habits. As some parents lack the time or ability to read with their children, children’s book publishers need to help parents supervise the process of their children’s reading through technical services. In recent years, there have been a number of mobile phone and tablet apps that have gone online with a teen mode to help parents correct their children’s media usage behavior. Integrated children’s book publishers can use existing cases for reference and incorporate parental control functions into their product design to provide technical safeguards for the reasonable use of such products.

## 7 Conclusion

Based on the UTAUT and HMSAM, this article studies the effects of curiosity, joy, performance expectancy, effort expectancy, social impact, and facilitating conditions on children’s adoption of integrated children’s books by constructing a structural equation model, and it concludes that children’s reading of integrated children’s books is simultaneously influenced by hedonic and utilitarian motivations.

At the level of hedonic motivation, both curiosity and joy have a positive impact on children’s reading intentions and behaviors, the effect of reading delight on reading intentions becomes more pronounced as children get older, and curiosity about integrated children’s books becomes less and less important. Moreover, a child’s home educational environment may be an important factor influencing their hedonic reading motivation: the parents’ level of education determines, to some extent, whether the child reads integrated children’s books out of joy or curiosity.

At the level of utilitarian motivation, performance expectancy, effort expectancy, and social impact all have an impact on children’s reading intentions. As for the two influencing factors of performance expectancy and effort expectancy, as children grow older, knowledge and technological thresholds are no longer the main constraints on children’s reading intentions, and parents’ perceptions of reading performance are transmitted to children more and more intensely, which is an important source of reading motivation. Nevertheless, in terms of social impact, it has always been a significant factor influencing children of any age group to choose integrated children’s books.

Based on the above verification, this article argues that children’s book publishers need to optimize the publishing path of integrated children’s products in three dimensions in the future. Firstly, publishers need to balance the concepts of ‘education oriented’ and ‘children oriented’ and integrate the two creative factors of ‘catering to nature’ and ‘cultivating habits.’ Secondly, publishers need to develop targeted technological empowerment strategies based on the different cognitive psychology of children in different age groups in order to satisfy the requirement of ‘Level Reading.’ Finally, publishers need to continuously innovate and optimize the service patterns of their children’s book products by introducing models such as parents accompanying studying and adult supervision to help children avoid the risk of poor reading. To be brief, the children’s book publishing industry needs to fully integrate various motivations and factors so that children can both gain knowledge and joy during reading.

## 8 Limitation

It must be acknowledged that this study has the following limitations:

Above all, due to the vast territory and large population of China, conducting rigorous probability sampling will add significant research costs. Therefore, this study adopted regional stratified sampling in an attempt to control for potential sampling bias. However, this may still affect the external validity of the study; thus, future research is required to reveal more evidence to validate the findings of this study.

Secondly, the respondents of this study were children aged 0–12 from 31 regions in China. Considering the ethical issues in research, the online questionnaire survey was conducted by child guardians, who answered the questions on the children’s behalf. Although we screened a considerable proportion of unqualified questionnaires by the answer–question time standard, there is potential for measurement bias in these answers. Henceforward, direct measurement methods such as offline observation and behavioral experiments can be used to avoid potential measurement bias and improve the reliability of research conclusions.

In addition, this study is mainly based on existing literature, theories, and the current situation of the Chinese digital publishing industry, using a top-down quantitative research path to inquire into the influence of utilitarian motivation and hedonic motivation on children’s adoption of integrated children’s books. However, there may be some other potential new influence factors that have not been fully contemplated. In the future, the understanding of this research problem can be expanded by observing children’s use of integrated children’s books and conducting qualitative analysis of interview data.

## Data availability statement

The raw data supporting the conclusions of this article will be made available by the authors, without undue reservation.

## Ethics statement

The studies involving humans were approved by Ethics Committee of School of Journalism and Communication, Henan University. The studies were conducted in accordance with the local legislation and institutional requirements. The participants provided their written informed consent to participate in this study. Written informed consent was obtained from the individual(s) for the publication of any potentially identifiable images or data included in this article.

## Author contributions

JC: Formal analysis, Supervision, Writing – original draft. JG: Data curation, Writing – review & editing. JY: Conceptualization, Resources, Writing – review & editing. LW: Conceptualization, Resources, Writing – review & editing. YB: Writing – review & editing.

## Footnotes

^1^In order to avoid confusing respondents because of the concept of ‘integrated children’s books’, the questionnaire referred to integrated children’s books as a ‘new-type of children’s books’ and illustrated four common types of products currently on the market, which are ‘children’s books that can be used with reading pens’, ‘reading machines which are equipped with children’s books’, ‘children’s books that can be scanned to listen to audio and watch videos’, and ‘children’s early learning machines and learning machines that can read children’s books’.

^2^All the structural model path coefficients in the text are standardized estimated values.
